# Synaptic Function and Modulation in Health and Disease

**DOI:** 10.3390/biomedicines14102272

**Published:** 2026-10-08

**Authors:** Jean-Marie C. Bouteiller

**Affiliations:** 1 Institute for Technology and Medical Systems, Keck School of Medicine, University of Southern California, Los Angeles, CA 90089, USA; jbouteil@usc.edu; 2 Alfred E. Mann Department of Biomedical Engineering, Viterbi School of Engineering, University of Southern California, Los Angeles, CA 90089, USA; 3 International Neurodegenerative Disorders Research Center (INDRC.cz), 16000 Prague, Czech Republic

Synapses are often described as specialized points of communication between neurons. Yet this seemingly simple definition conceals a remarkably dynamic and complex biological system. Synaptic efficacy depends on the coordinated organization of presynaptic release machinery, postsynaptic receptors and scaffolding proteins, neuromodulatory signals, metabolic support, glial interactions, and the history of neuronal activity. These elements operate across many spatial and temporal scales, allowing synapses to adapt to changing physiological demands while also making them vulnerable to disturbances associated with stress, aging, and neurological diseases.

The Special Issue “Synaptic Function and Modulation in Health and Disease” brings together contributions that illustrate this complexity from complementary perspectives [1,2,3,4,5,6]. The collection includes experimental studies, computational modeling, and reviews addressing inhibitory and excitatory neurotransmission, neuromodulation, glial regulation, metabolism, and synaptic dysfunction in neurodegeneration. Taken together, these contributions reinforce an increasingly important view of the synapse: it is not a static connection, but a dynamic and context-dependent biological unit whose computational properties emerge from interactions across multiple levels of organization.

## 1. Synaptic Receptors Are Dynamic Components of a Larger Signaling System

An important contribution to this Special Issue is the study by Wei et al. [1], which examines the regulation of glutamate and GABA release by α2A-adrenoceptors in the rat prefrontal cortex (PFC). α2A-adrenoceptors are classically viewed as presynaptic autoreceptors that regulate norepinephrine release. The study demonstrates, however, that these receptors are also present at glutamatergic and GABAergic terminals in the PFC. Activation by guanfacine reduced the frequency, but not the amplitude, of miniature excitatory and inhibitory postsynaptic currents; these effects persisted after depletion of presynaptic norepinephrine vesicles. These findings identify α2A-adrenoceptors as functional heteroreceptors capable of modulating both excitatory and inhibitory transmitter release. They therefore provide an additional mechanism through which noradrenergic signaling can regulate the balance of activity in cortical circuits.

This work highlights a broader principle: neuromodulators cannot necessarily be understood by considering only their classical targets. A receptor’s functional impact depends on its precise subcellular localization and on the identity of the terminal in which it operates. Such heterosynaptic mechanisms may be particularly important in brain regions such as the PFC, where appropriate control of excitation and inhibition is essential for cognitive functions.

A complementary perspective is provided by Kiss et al. [2], who revisit the mechanisms governing clustering of inhibitory glycine and GABA A receptors. Their review emphasizes that receptor clustering is regulated by multiple mechanisms, including lateral diffusion, anchoring, trafficking, and neurotransmitter-dependent signaling. A particularly interesting distinction emerges between glycine and GABA. Evidence accumulated over more than two decades suggests that glycine receptor activation is important for glycine receptor (GlyR) cluster formation, whereas initial GABA A receptor clustering can occur in the absence of ligand-dependent activation. At the same time, neurotransmitter release can modulate the size and organization of existing receptor clusters.

These observations are especially relevant in light of recent advances in imaging synapses at nanometer-scale resolutions. Super-resolution microscopy and cryo-electron tomography are revealing that postsynaptic receptors, scaffolding proteins, and adhesion molecules are arranged into highly organized subsynaptic domains rather than being distributed uniformly [2]. Activity-dependent changes in this nanoarchitecture may therefore represent an important layer of synaptic plasticity that is not captured by conventional measurements of synapse number or receptor abundance.

## 2. Bridging Molecular Complexity and Large-Scale Computation

The complexity of synaptic receptors creates a major challenge for computational neuroscience. NMDA receptors provide an excellent example because their responses depend on voltage, magnesium block, co-agonists, activation history, receptor kinetics, and subunit composition. Beyond their physiological roles in synaptic plasticity, altered NMDA receptor signaling is implicated in multiple neurodegenerative disorders and continues to attract interest as a potential therapeutic target [3]. Although computationally efficient, exponential synaptic models cannot reproduce many of these nonlinear features.

Pham and Bouteiller [4] address this challenge by combining experimentally constrained mechanistic modeling with a computationally efficient data-driven representation. They optimized a nine-state kinetic model of GluN1/GluN2A NMDA receptor dynamics against experimental observations and subsequently used this model to construct a look-up-table synapse model. The resulting model reproduced experimentally observed temporal responses and frequency-dependent potentiation while retaining a substantially smaller computational footprint than detailed kinetic descriptions.

The significance of this contribution extends beyond NMDA receptors themselves. Large-scale computational models require a difficult compromise between biological realism and computational tractability. The look-up-table approach they propose demonstrates how experimental data can be used to construct reduced representations that preserve relevant nonlinear behavior without explicitly simulating every molecular state. Previous large-scale simulations using this modeling framework reported a 38-fold increase in computational speed relative to a detailed 15-state kinetic NMDA receptor model, while preserving nonlinear synaptic dynamics that are lost with simpler exponential representations [4].

This kind of multiscale modeling is becoming increasingly important as neuroscience moves toward more detailed simulations of neuronal populations and brain circuits. Rather than viewing mechanistic and reduced models as competing alternatives, they can be regarded as complementary descriptions operating at different scales.

## 3. The Synapse Does Not Operate in Isolation

Two contributions in this Special Issue broaden the concept of synaptic modulation beyond neuronal signaling itself. Rábago-Monzón et al. [5] review the reciprocal relationship between chronic stress and sleep disruption, emphasizing the roles of astrocytes and microglia. Their analysis connects stress-induced activation of the hypothalamic–pituitary–adrenal axis with changes in glial function, neuroinflammation, adenosine signaling, glutamate homeostasis, glymphatic clearance, and synaptic pruning. The resulting picture is a self-reinforcing cycle in which stress disrupts sleep, altered glial function compromises synaptic homeostasis, and impaired sleep further amplifies pathological processes.

This perspective is consistent with a broader transformation in the field. Astrocytes are increasingly recognized as active regulators of synaptic structure and function rather than merely as supporting cells. Recent work on the tripartite synapse indicates that astrocytic processes can dynamically alter their structural relationships with synapses and that abnormalities in astrocyte–synapse interactions are associated with both neuropsychiatric and neurodegenerative disorders [6].

Metabolism represents another layer of synaptic regulation. Targett et al. [7] reviewed evidence linking diet, systemic metabolism, and brain energy metabolism to synaptic function and neurodegeneration. Their analysis emphasizes that metabolic influences may act both locally within the CNS and indirectly through systemic insulin resistance, dyslipidemia, and inflammation. Importantly, the authors distinguish between human observational evidence and mechanistic evidence obtained from animal and cellular models, highlighting the difficulty of translating experimental dietary paradigms into human disease.

This subject is particularly timely because emerging work is challenging several traditional assumptions concerning neuronal metabolism. Astrocyte–neuron metabolic coupling, neuronal use of lipid-derived substrates, and brain insulin signaling are increasingly being recognized as potential determinants of synaptic health [7]. Thus, synaptic physiology cannot be completely separated from the metabolic environment in which neurons and glia operate.

## 4. From Synaptic Modulation to Synaptic Vulnerability

The final contribution, by Argibay et al. [8], focuses on the role of α-synuclein in the prefrontal cortex (PFC) and provides a disease-oriented perspective on the preceding themes. α-Synuclein has traditionally been studied primarily in the context of dopaminergic dysfunction in Parkinson’s disease. The review emphasizes, however, that α-synuclein is also an important regulator of synaptic vesicle dynamics in cortical circuits and participates in the regulation of glutamatergic and GABAergic transmission. Its pathological conversion into oligomeric and aggregated forms is associated with synaptic dysfunction, dendritic spine abnormalities, loss of synaptic markers, and impaired cortical plasticity.

Importantly, the evidence summarized in this review suggests that synaptic pathology may develop before extensive neuronal loss. Experimental models in which α-synuclein pathology is introduced into the PFC produce abnormalities in cortico-amygdala transmission and behavioral deficits, whereas clinical studies indicate alterations in cortical glutamatergic markers in Parkinson’s disease [8]. This reinforces the concept that dysfunction of synaptic connectivity may be an early and clinically meaningful component of neurodegenerative disease rather than simply a consequence of late-stage neuronal degeneration.

Recent research across dementias similarly suggests that synapses differ markedly in their susceptibility to pathology. Some synapses are preferentially lost, whereas others remain relatively resilient, raising the possibility that understanding the molecular basis of synaptic resilience may be as important as understanding mechanisms of synaptic degeneration [9].

## 5. Looking Ahead: Toward an Integrated View of Synaptic Health

As highlighted above, a major strength of this Special Issue is the convergence of several traditionally separated areas of neuroscience. Receptor kinetics, postsynaptic organization, neuromodulation, glial biology, metabolism, computational modeling, and neurodegeneration are not independent subjects. They describe different components of the same adaptive system.

Recent work extends this integrative, multiscale perspective to astrocyte-mediated plasticity, linking astrocytic Ca^2+^ signaling, gliotransmission, local translation, and perisynaptic structural dynamics to synaptic efficacy, circuit computation, learning, and memory [10].

Several directions seem particularly important for the future of the field. First, synaptic biology increasingly needs to be studied with cell-type, compartment, and even nanodomain specificity. Recent advances in super-resolution and volumetric imaging, single-cell and spatial transcriptomics, and selective manipulation of glial populations are making such analyses increasingly feasible [2,5,9,10].

Second, molecular measurements need to be connected more systematically to circuit-level function and behavior. Computational models provide one possible bridge, allowing detailed experimental observations to be translated into network-level hypotheses [4]. Such models will become more valuable as they incorporate increasingly heterogeneous synapses, neuromodulatory states, and interactions with glial and metabolic processes.

Third, disease research should move beyond the question of why synapses are lost toward the complementary question of why particular synapses survive. The emerging concept of synaptic vulnerability and resilience offers a promising framework for identifying disease-specific mechanisms and, potentially, therapeutic strategies aimed at preserving functional connectivity rather than simply correcting a single molecular defect [9].

Finally, the increasing recognition of the synapse as a multicellular and metabolically integrated structure has therapeutic implications. Broadly suppressing excitatory or inhibitory transmission is unlikely to reproduce the nuanced regulation achieved by healthy neural circuits. Future interventions may instead need to target specific receptor populations, trafficking mechanisms, neuromodulatory pathways, glial interactions, or metabolic processes, while preserving the adaptive plasticity that makes synapses functional [1,2,5,7]. Achieving this level of therapeutic precision will require a comprehensive understanding of the multiple processes that together shape and modulate synaptic health and function.

Across these contributions, a common principle emerges: synaptic function is inherently context dependent. Receptor dynamics depend on activation history and molecular composition; transmitter release depends on neuromodulatory state and terminal identity; synaptic organization depends on activity and cellular interactions; and synaptic resilience depends on metabolic, glial, and pathological context. Understanding the synapse therefore increasingly requires integration across molecular, cellular, circuit, and systemic scales.

The contributions assembled in this Special Issue illustrate how far the concept of “synapse” has evolved. From receptor state transitions measured in milliseconds to changes in synaptic architecture occurring over days, and from molecular mechanisms to cognitive and behavioral consequences, synaptic function emerges as a complex multiscale phenomenon. Understanding this complexity will be essential not only for explaining how healthy neural circuits adapt, learn, and maintain stability, but also for identifying the earliest and most tractable points at which disease-related synaptic dysfunction can be prevented or reversed.

## Abbreviations

The following abbreviations are used in this manuscript.

PFC	Prefrontal cortex
CNS	Central nervous system
GlyR	Glycine receptor
Ca^2+^	Calcium

## References

[1] Wei Y., Jiao Y., He X., Tao X., Li B., Zhang X. (2025). Heterosynaptic Regulation of α2A-Adrenoceptors on Glutamate/GABA Release in the Prefrontal Cortex of Rats. Biomedicines.

[2] Kiss E., Kirsch J., Kuhse J., Kins S. (2025). Is the Activation of the Postsynaptic Ligand Gated Glycine- or GABAA Receptors Essential for the Receptor Clustering at Inhibitory Synapses?. Biomedicines.

[3] Zhang K., Wen M., Nan X., Zhao S., Li H., Ai Y., Zhu H. (2025). NMDA Receptors in Neurodegenerative Diseases: Mechanisms and Emerging Therapeutic Strategies. Front. Aging Neurosci..

[4] Pham D.-T.J., Bouteiller J.-M.C. (2025). Experimentally Constrained Mechanistic and Data-Driven Models for Simulating NMDA Receptor Dynamics. Biomedicines.

[5] Rábago-Monzón Á.R., Osuna-Ramos J.F., Armienta-Rojas D.A., Camberos-Barraza J., Camacho-Zamora A., Magaña-Gómez J.A., De la Herrán-Arita A.K. (2025). Stress-Induced Sleep Dysregulation: The Roles of Astrocytes and Microglia in Neurodegenerative and Psychiatric Disorders. Biomedicines.

[6] Badia-Soteras A., Mak A., Blok T.M., Boers-Escuder C., van den Oever M.C., Min R., Smit A.B., Verheijen M.H.G. (2025). Astrocyte-Synapse Structural Plasticity in Neurodegenerative and Neuropsychiatric Diseases. Biol. Psychiatry.

[7] Targett I.L., Hancock J.T., Craig T.J. (2026). Diet, Metabolism and Synaptic Function: Integrating Evidence Across Models in Neurodegeneration Research. Biomedicines.

[8] Argibay U., Sancho-Alonso M., Yanes-Castilla C., Jericó-Escolar J., Paz V., Ruiz-Bronchal E., Miquel-Rio L., Bortolozzi A. (2026). Role of α-Synuclein in the Prefrontal Cortex: From Physiological Synaptic Modulation to Synaptic Failure in Parkinson’s Disease. Biomedicines.

[9] Taddei R.N., Duff K.E. (2025). Synapse Vulnerability and Resilience across the Clinical Spectrum of Dementias. Nat. Rev. Neurol..

[10] Yamamoto M., Takano T. (2025). Astrocyte-Mediated Plasticity: Multi-Scale Mechanisms Linking Synaptic Dynamics to Learning and Memory. Cells.

